# The Prevalence of Antibodies against Wheat and Milk Proteins in Blood Donors and Their Contribution to Neuroimmune Reactivities

**DOI:** 10.3390/nu6010015

**Published:** 2013-12-19

**Authors:** Aristo Vojdani, Datis Kharrazian, Partha Sarathi Mukherjee

**Affiliations:** 1 Immunosciences Lab., Inc., 822 S. Robertson Blvd., Ste. 312, Los Angeles, CA 90035, USA; 2 Department of Clinical Sciences, Bastyr University California, 4106 Sorrento Valley Blvd, San Diego, CA 92121, USA; E-Mail: datis56@gmail.com; 3 Department of Mathematics, Boise State University, 1910 University Dr., Boise, ID 83725, USA; E-Mail: parthamukherjee@boisestate.edu

**Keywords:** antibodies, wheat proteins, milk proteins, neuroimmune

## Abstract

The aim of this study was to look for the presence of IgG, IgM, and IgA antibodies against two widely consumed foods, wheat and milk, in a relatively large number of specimens. As wheat, milk, and their antigens have been found to be involved in neuroimmune disorders, we measured the co-occurrence of their antibodies against various neural antigens. We assessed the reactivity of sera from 400 donors to wheat and milk proteins, GAD-65, cerebellar, MBP, and MOG. Statistical analysis showed significant clustering when certain wheat and milk protein antibodies were cross-referenced with neural antibodies. Approximately half of the sera with antibody elevation against gliadin reacted significantly with GAD-65 and cerebellar peptides; about half of the sera with elevated antibodies against α + β-casein and milk butyrophilin also showed antibody elevation against MBP and MOG. Inhibition studies showed that only two out of four of the samples with elevated cerebellar or MOG antibodies could be inhibited by gliadin or α + β-casein, confirming individual variation in epitope recognition. We conclude that a subgroup of blood donors, due to a breakdown in immunological tolerance, may react and produce significant levels of antibodies (*p*-values less than 0.05) against wheat and milk antigens that cross-react with different neural antigens, which may have broader implications in the induction of neuroimmune reactivities.

## 1. Introduction

Given the variety and abundance of foods available in the marketplace, eating is an enjoyable life experience. For some individuals, however, consuming certain foods can be a pathological, possibly life-threatening event. This is why the Roman philosopher Lucretius said, many years ago, that one man’s food may be another man’s poison. Such is the case with food allergies and sensitivities [1]. The discussion about food allergy and sensitivity, in particular with regards to gluten and casein, and associated health problems has grown over the last decade. Classical food allergies are becoming more prevalent and recognized in our society. It is estimated that 5%–6% of children and 3%–4% of adults may have IgE-mediated or immediate type hypersensitivity to various food antigens [2,3]. A meta-analysis of 57 articles from different countries examined the prevalence of food allergies by using different criteria. Depending on food antigens, location, and gender, the authors found marked heterogeneity between studies [4,5,6,7,8].

The big eight; milk, wheat, eggs, peanuts, fish, crustacean, tree nuts, and soy beans, are thought to account for more than 90% of all IgE-mediated food allergies in the USA, as well as on a worldwide basis [1,5]. Estimates for food sensitivities that are not IgE-mediated are more difficult to determine, since percentages may vary from one food antigen to another, and even from gender to gender and location to location [7,8]. For example, reaction to various wheat proteomes could be involved in three distinct conditions called wheat allergy, celiac disease (CD) and non-celiac gluten sensitivity (NCGS) [9,10,11]. In these conditions, the reaction to gluten is mediated by both cellular and humoral immune responses, resulting in the presentation of different symptomatologies. In fact, in wheat allergy a specific sequence of gliadin peptides cross-links two IgE molecules on the surface of mast cells and basophils that trigger the release of mediators, such as histamines and leukotrienes [12].

Celiac disease is an autoimmune condition with known genetic makeup and environmental triggers, such as gliadin peptides. CD affects between 1% and 2% of the general population. Markers for confirming a diagnosis of this disorder are IgA against native, deamidated gliadin peptides, and IgA anti-tissue transglutaminase (tTg) autoantibody. In comparison with CD, NCGS may affect 6% to 7% of the population, [13,14]. According to two articles published in 2010 and 2011 by Sapone *et al*. [13,14], symptoms in GS may resemble some of the gastrointestinal symptoms that are associated with CD or wheat allergy, but it is emphasized that objective diagnostic tests for nonceliac gluten sensitivity are currently missing [13,14].

Very recently, in a case series study conducted in our laboratory, we attempted to use ELISA methodology to differentiate between CD and NCGS [15]. The results brought us to contemplate that continuous exposure to environmental factors, such as wheat antigen-induced inflammation for a prolonged period of time, may result in inflammatory bowel disease or Crohn’s disease.

Indeed, it has been shown that both CD and NCGS can result in many autoimmune conditions, including type 1 diabetes, arthritis, thyroiditis, and even neuroautoimmune conditions, such as gluten ataxia and multiple sclerosis (MS) [15,16,17,18].

Nutritionists through the years seem to agree that milk is one of the most basic necessities of a healthy diet. However, unfortunately, cow’s milk proteins are the most common food allergens affecting young infants and some children and adults [19]. Major allergenic proteins of milk are α-casein, β-casein, κ-casein, and β-lactoglobulin. In addition to IgE-mediated cow’s milk allergy there is increasing awareness that early consumption of cow’s milk may be a risk for the development of autoimmune diseases such as celiac disease, Crohn’s disease, Behçets disease, MS, mild rheumatoid arthritis in rabbits, and type 1 diabetes in humans [20,21,22,23,24,25]. These findings are supported by the detection of significantly higher levels of IgG and IgA antibodies in disease sufferers compared to normal controls. In these studies it was concluded that active immune response against cow’s milk proteins plays a putative role in the pathogenesis of autoimmune disorders [7,20,21,22,23,24,25]. Despite these elevations in the IgG and IgA antibodies against wheat and milk proteins in blood samples of patients with various autoimmune disorders, the immunopathological role of these antibodies were not examined in the context of celiac disease in the IgE and non-IgE mediated reactivities [9,10,26,27,28,29]. Furthermore, in the non-IgE-mediated delayed wheat and milk reactivities, only IgG and IgA were studied and the role of IgM was completely ignored [15,26,30]. Therefore, the aims of this study were threefold: (1) to examine the frequency of IgG-, IgA- and IgM-specific antibodies produced against wheat and milk proteins; (2) to examine the co-occurrence of these antibodies in sera with antibody elevation against glutamic acid decarboxylase (GAD-65), myelin basic protein (MBP), cerebellar peptide, and myelin oligodendrocytes glycoprotein (MOG); and (3) to discuss the mechanism of action of these antibodies in various neuroautoimmune reactivities in a subgroup of healthy controls.

## 2. Experimental Section

### 2.1. Materials and Methods

#### 2.1.1. Blood Samples

Blood samples from 400 blood donors (181 males and 219 females, cross-spectrum of the population, mixture of Caucasians, Hispanics, and African-Americans, aged 18 and older) were purchased from Innovative Research Inc. (Southfield, MI, USA). Prior to shipping, each blood sample was tested according to FDA guidelines for the detection of hepatitis B surface antigen, antibodies to HIV, antibodies to hepatitis C, HIV-1 RNA, hepatitis C RNA, and syphilis. All units yielded non-reactive/negative results for each test performed. No medical examinations or additional lab tests were conducted to otherwise determine the health status of the donors. The diseased state sera from patients with neuroautoimmune disorders and celiac disease were purchased from The Binding Site (Birmingham, UK). Blood samples were kept at −40 °C until used for antibody measurements.

#### 2.1.2. Proteins and Peptides

Wheat, cow’s milk, α-casein, β-casein, and MBP were purchased from Sigma-Aldrich (St. Louis, MO, USA).

γ-Gliadin 15-mer NH2-PQQPQQSFPQQQQPA-OH,
γ-gliadin 18-mer NH2-LPFPEQPEQPFPQPEQPQ-OH,
α-gliadin 33-mer peptide NH2-LQLQPFPQLPYPQPQLPYPQPQLYPQPQPF-OH,
milk butyrophilin peptide NH2-APFDVIGPQEPILAVVGEDA-OH,
GAD-65 peptide NH2-TLEDNEERMSRLSKVAPVIKARMMMEYGT-OH,
cerebellar peptide NH2-FLEDVPWLEDVDFLEDVPLLED-OH,
and MOG peptide NH2- IGEGKVTLRIRN-OH HPLC grade were synthesized by Bio-synthesis Inc. (Lewisville, TX, USA).

#### 2.1.3. Detection of Antibodies by ELISA

Antigens and peptides were dissolved in PBS or methanol at a concentration of 1.0 mg/mL, then diluted 1:100 in 0.1 M carbonate-bicarbonate buffer, pH 9.5, and 100 μL were added to each well of a polystyrene flat bottom ELISA plate. Plates were incubated overnight at 4 °C and then washed three times with 200 μL Tris-buffered Saline (TBS) containing 0.05% Tween 20, pH 7.4. The non-specific binding of immunoglobulins was prevented by adding a mixture of 1.5% bovine serum albumin (BSA) and 1.5% gelatin in TBS, and incubated overnight at 4 °C. Plates were washed as described above, and then serum samples diluted 1:100 in 0.1 M PBS Tween containing 2% BSA were added to duplicate wells and incubated for 1 h at room temperature. Sera from patients with celiac disease and neuroimmune disorders, with known high titers of antibodies, were used as positive controls. Plates were washed, and then alkaline phosphatase goat anti-human IgG, IgM or IgA F(ab′)2 fragments (KPI, Gaithersburg, MD, USA) optimal dilution of 1:400–1:2000 in 1% HSA-TBS was added to each well; plates were incubated for an additional 1 h at room temperature. After washing five times with TBS-Tween buffer, the enzyme reaction was started by adding 100 μL of paranitrophenylphosphate (PNPP) in 0.1 mL diethanolamine buffer 1 mg/mL containing 1 mM MgCl_2_ and sodium azide pH 9.8. The reaction was stopped 45 min later with 50 μL of 1 N NaOH. The optical density (OD) was read at 405 nm by means of a microtiter reader. To detect non-specific binding, several control wells contained all reagents except human serum, or wells were coated with different tissue antigens, such as liver and kidney. Human serum and all other reagents were added and used in each assay. This assay was applied to wheat antigen α-gliadin 33-mer, γ-gliadin 15-mer and 18-mer, GAD-65 and cerebellar coated plates, followed by measuring antibodies against milk, α + β-casein, milk butyrophilin, MBP and MOG simultaneously.

#### 2.1.4. Absorption of Sera with Different Antigens

Four different sera containing high levels of IgG, IgM, or IgA antibodies against cerebellar and GAD-65 were used in inhibition studies. In a different test tube, 1 mL of 1:100 diluted serum sample was pre-incubated with 100 μL diluent containing 100 μg of wheat antigens, gliadin peptide, GAD-65, or cerebellar peptide. Similarly, four different sera containing high levels of IgG, IgM or IgA antibodies against MBP and MOG diluted 1:100 were put in different test tubes. These sera were pre-incubated with 100 μL diluent containing 100 μg of either HSA, milk, α + β-casein and milk butyrophilin. After mixing, the tubes were kept for 1 h in a 37 °C water bath followed by 4 h incubation at 4 °C and then centrifuged at 3000 g for 10 min. The supernatant was used for measuring antibody levels against cerebellar peptide and MOG and comparing the ELISA OD of unabsorbed to absorbed sera.

#### 2.1.5. Coefficients of Intra- and Inter-Assay Variation

Coefficients of intra-assay variation were calculated by running five samples eight times within a single assay. Coefficients of inter-assay variation were determined by measuring the same samples in six consecutive assays. This replicate testing established the validity of the ELISA assays, determined the appropriate dilution with minimal background, and detected serum IgG, IgM, and IgA against different antigens. Coefficients of intra- and inter-assay variations for IgG, IgM, and IgA against all tested antigens and peptides were less than 10%.

#### 2.1.6. Statistical Methods Used in the Data Analysis

At first, we calculated the percentages of specimens that were positive (% seropositivity) for each isotype antibody that exhibited elevation by at least 2 standard deviations (2 SD) above the mean against wheat, milk proteins, and various neural tissue antigens. Next, we performed Pearson’s Chi-squared tests to investigate the statistical significance among the differences between the percentages of elevation in each isotype of antibodies against wheat and tissue proteins. We then calculated Pearson’s correlation coefficient between each isotype (lgG, lgA, and lgM) of food protein (wheat, α-gliadin 33-mer, γ-gliadin, cow’s milk, casein, milk butyrophilin) and similar isotype of brain protein (GAD-65, cerebellar, MBP, and MOG). Subsequently, we performed simple regression analysis between each of those combinations, and calculated their *p*-values. Simple regression analysis between each pair of food proteins within each isotype was also performed. Finally, we carried out a two-way cluster analysis of the Pearson’s correlation coefficients between the food protein antibodies and the neural antibodies. We performed all statistical tests and the two-way cluster analysis in the statistical software “R”.

## 3. Results

**Figure 1:**
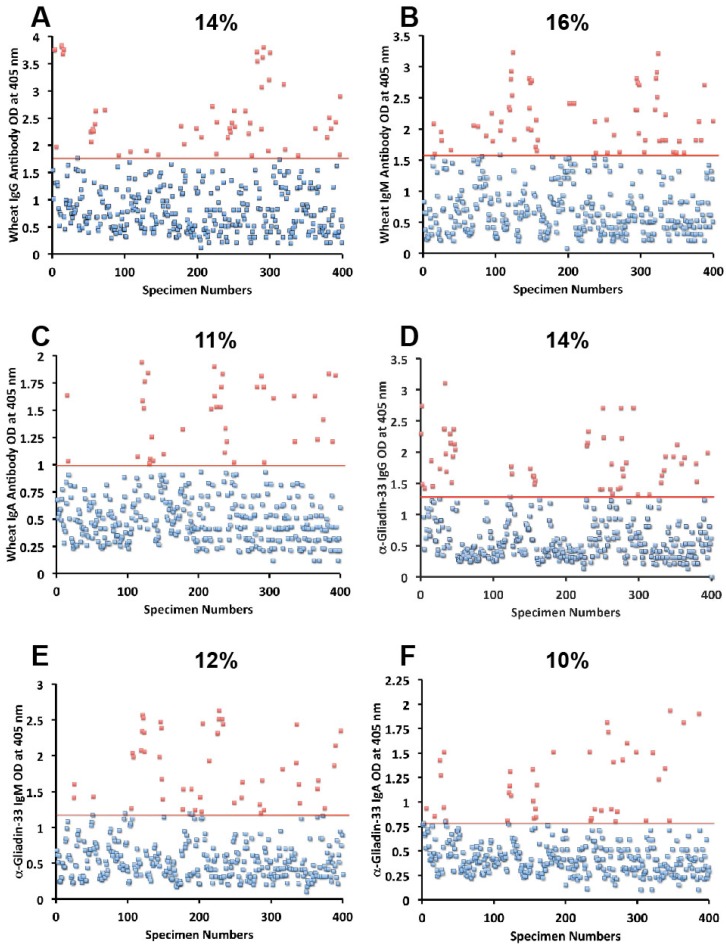
Results for wheat and α-gliadin-33 expressed as OD at 405 nm in the form of scattergrams with percentages of elevation. (**A**) Wheat IgG; (**B**) Wheat IgM; (**C**) Wheat IgA; (**D**) α-gliadin-33 IgG; (**E**) α-gliadin-33 IgM; (**F**) α-gliadin-33 IgA.

**Figure 2:**
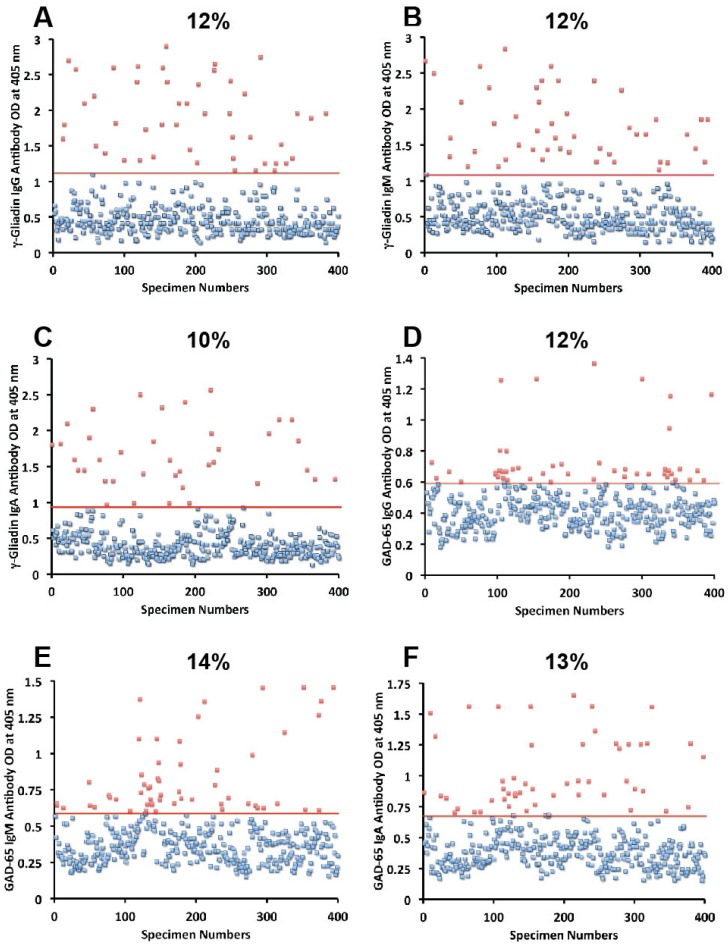
Results for γ-gliadin and GAD-65 expressed as OD at 405 nm in the form of scattergrams with percentages of elevation. (**A**) γ-gliadin IgG; (**B**) γ-gliadin IgM; (**C**) γ-gliadin IgA; (**D**) GAD-65 IgG; (**E**) GAD-65 IgM; (**F**) GAD-65 IgA.

**Figure 3:**
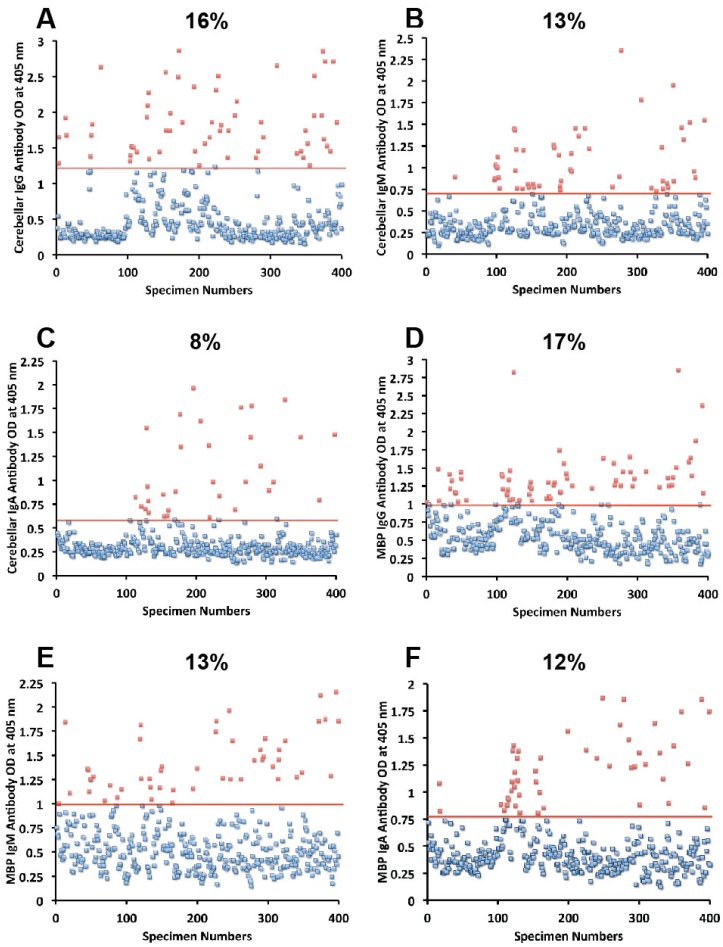
Results for cerebellar and MBP expressed as OD at 405 nm in the form of scattergrams with percentages of elevation. (**A**) Cerebellar IgG; (**B**) Cerebellar IgM; (**C**) Cerebellar IgA; (**D**) MBP IgG; (**E**) MBP IgM; (**F**) MBP IgA.

**Figure 4:**
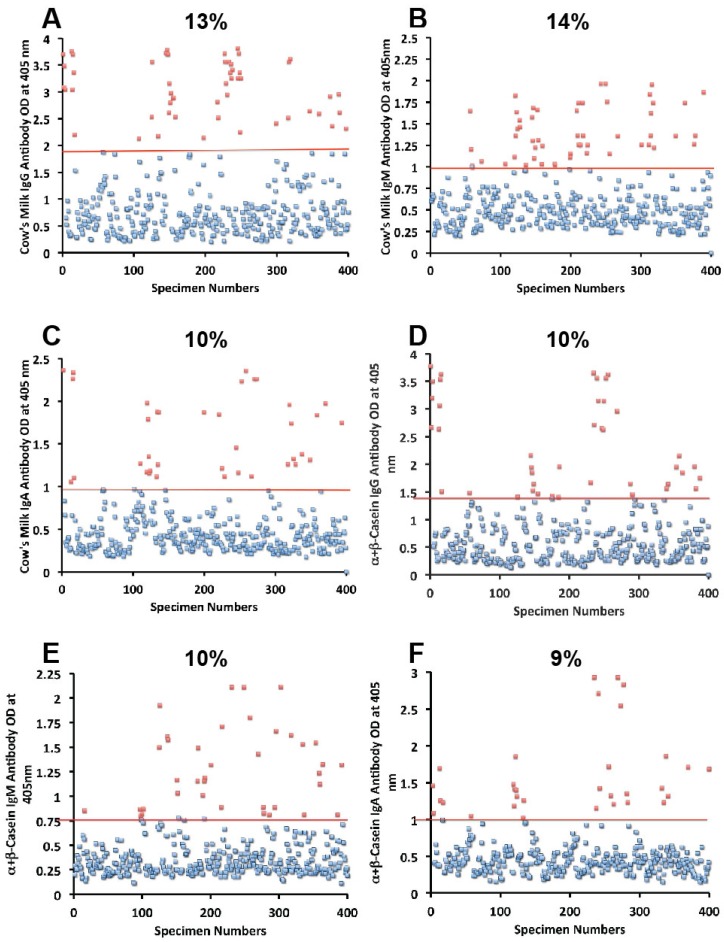
Results for cow’s milk and α + β-casein expressed as OD at 405 nm in the form of scattergrams with percentages of elevation. (**A**) Cow’s milk IgG; (**B**) Cow’s milk IgM; (**C**) Cow’s milk IgA; (**D**) α + β-casein IgG; (**E**) α + β-casein IgM; (**F**) α + β-casein IgA.

**Figure 5:**
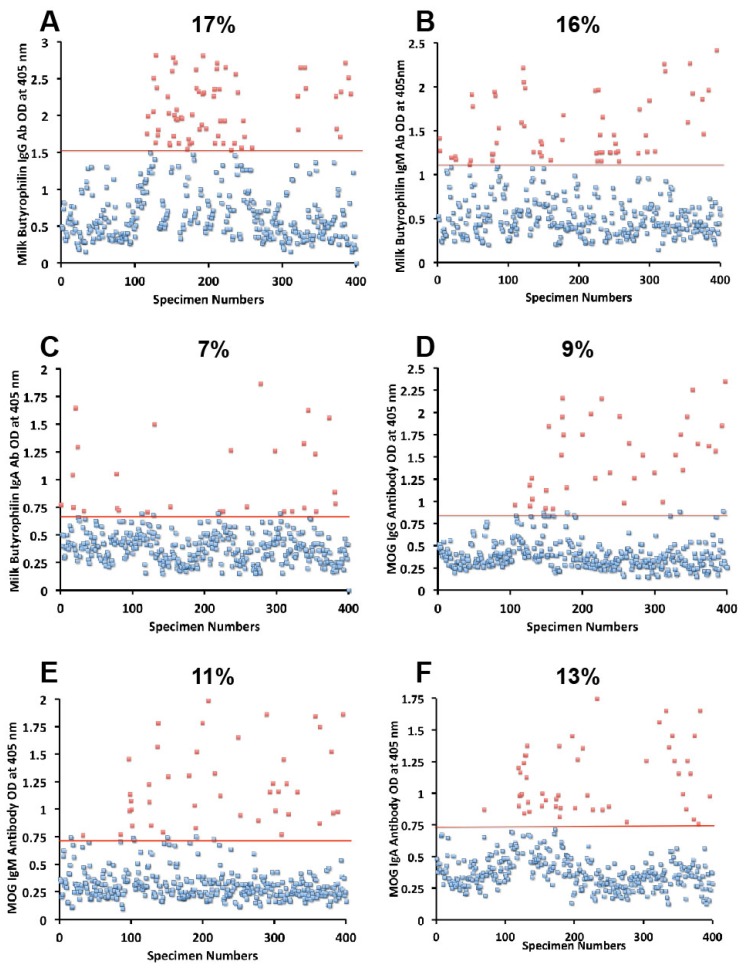
Results for milk butyrophilin and MOG expressed as OD at 405 nm in the form of scattergrams with percentages of elevation. (**A**) Milk butyrophilin IgG; (**B**) Milk butyrophilin IgM; (**C**) Milk butyrophilin IgA; (**D**) MOG IgG; (**E**) MOG IgM; (**F**) MOG IgA.

Sera from 400 blood donors were measured for the simultaneous presence of IgG, IgM, and IgA antibodies against wheat, α-gliadin 33-mer, γ-gliadin 15-mer and 18-mer, milk, α + β-casein, milk butyrophilin, GAD-65, cerebellar, MBP, and MOG. Results expressed as OD at 405 nm in the form of scattergrams are shown in Figure 1, Figure 2, Figure 3, Figure 4 and Figure 5. The OD for IgG antibody levels against wheat ranged from 0.1 to 3.8 with mean value of 1.0. At two standard deviations above the mean or OD of 1.8, 14% of individuals exhibited IgG antibody against wheat proteins (Figure 1A). The mean OD of IgM anti-wheat was 0.92 and % elevation was 16 (Figure 1B), while for wheat IgA antibody the mean OD was 0.6 with only 11% elevation (Figure 1C). Levels of these antibodies against α-gliadin 33-mer peptide expressed by ODs were from 0.1 to 3.1 with mean 0.74 (Figure 1D) for IgG, 0.1–2.6 with mean 0.68 for IgM (Figure 1E), and from 0.1 to 1.9 with mean 0.49 for IgA (Figure 1F). The percentage elevation of antibodies against α-gliadin 33-mer were 14, 12, and 10 for IgG, IgM, and IgA, respectively. Regarding the γ-gliadin antibody, while the ODs varied from 0.14 to 2.9 at 2 SD above the mean, 12% for IgG, 12% for IgM, and 10% for IgA of the specimens exhibited antibody elevation (Figure 2A–C). The distribution of milk-, α + β-casein- and butyrophilin-specific antibody levels were as follows: for cow’s milk, elevations were IgG 13%, IgM 14%, and IgA 10%; (Figure 4A–C). For α + β-casein, elevations for IgG and IgM were 10%, while IgA was 9% (Figure 4D–F). And for milk butyrophilin, elevations were IgG 17%, IgM 16%, and IgA 7% (Figure 5A–C).

### 3.1. Measurement of Antibodies against Neural Antigens

Concomitant with the increase of IgG, IgM, and IgA antibodies against wheat, α- and γ-gliadin, milk, α + β-casein, and milk butyrophilin, in a small subgroup of individuals we observed a significant increase in GAD-65 (Figure 2D–F), cerebellar peptide (Figure 3A–C), MBP (Figure 3D–F), and MOG antibodies (Figure 5D–F). The percentage of elevation in antibodies against GAD-65, cerebellar, MBP and MOG varied from 9% to 17% for IgG, 11%–14% for IgM, and 8%–13% for IgA. Fifty-three out of 400 specimens (13%) showed IgG elevation against milk, and 68 or 17% had IgG elevation against MBP; about one third of these specimens positive for milk or MBP showed simultaneous elevation in IgG antibody against α + β-casein + MBP.

### 3.2. Absorption of Sera with High Titer of Antibody against Cerebellar or MOG with Specific and Non-Specific Antigens

**Figure 6:**
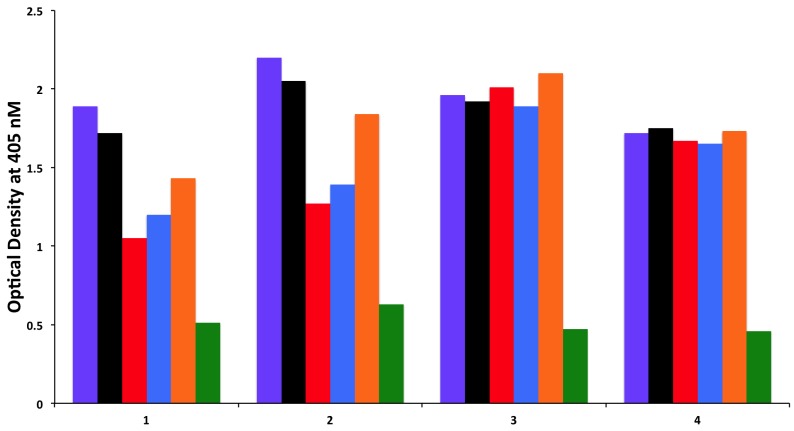
Inhibition of immune reaction of sera containing high levels of IgG, IgM and IgA antibody against cerebellar before 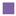 and after absorption with HSA 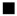, wheat 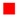, gliadin peptide 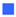, GAD-65 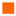 and cerebellar peptide 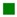.

**Figure 7:**
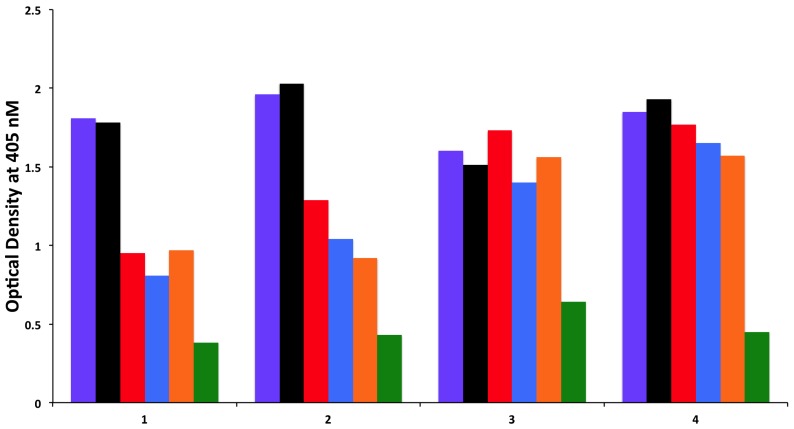
Inhibition of immune reaction of sera containing high levels of IgG, IgM and IgA antibody against MOG both before 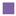 and after absorption with HSA 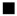, milk 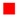, milk butyrophilin 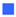, α + β-casein 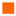, and MOG 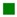.

To examine whether antibodies detected against cerebellar are cross-reacting with wheat proteins and antibodies detected against MOG are cross-reacting with milk proteins, we performed an absorption study with non-specific antigens, specific antigens, and possible cross-reactive antigens. Four different sera with high levels of antibodies against cerebellar were subjected to the absorption study with HSA as a non-specific antigen, cerebellar peptide as a specific antigen, and wheat antigen, gliadin, and GAD-65 peptides as possible cross-reactive antigens. In all four of these sera, the high titers of cerebellar antibody were inhibited by more than 70% by the addition of cerebellar peptide. However, it was only in serum numbers 1 and 2 that the antibody levels were inhibited by 38%, 34%, and 23%, respectively, after the addition of wheat, gliadin, and GAD-65, resulting in *p* values of 0.026 for wheat, 0.036 for gliadin, and 0.13 for GAD-65. Very similar results were obtained when sera with high levels of MOG antibody were subjected to absorption with HSA as a non-specific antigen, MOG peptide as a specific antigen, and milk, milk butyrophilin, and α + β-casein peptides as cross-reactive antigens. In all four of these sera, the high titers of MOG antibody were inhibited by more than 70% after the addition of MOG to the mixture. As was the case with the cerebellar sera, it was only in serum numbers 1 and 2 that the antibody levels were inhibited by about 40% by the addition of milk, α + β-casein and milk butyrophilin peptides to the mixture, resulting in *p* values of 0.049 for milk, 0.014 for milk butyrophilin, and 0.016 for α + β-casein. Inhibition with the same antigens for the third serum was 20%–25%, and no inhibition at all was observed with the fourth serum (Figure 6 and Figure 7).

Sample numbers

Sample numbers

### 3.3. Statistical Analyses of the Data for Investigating Association between the Food Proteins and the Brain Proteins

Next, we tested whether there are significant associations between the elevations of lgG, lgA, and lgM isotypes of GAD-65, cerebellar, MBP, and MOG with similar isotypes of wheat, gliadin-33, γ-gliadin, cow’s milk, casein, and milk butyrophilin. We fitted simple linear regression models between each such pairs, and calculated the *R*^2^ values and the *p*-values. The summary of the results is presented in the Table 1, Table 2 and Table 3. From the tables we see that several food proteins significantly elevates similar isotypes of some brain proteins. Specifically, considering statistically significant elevations only, we found that wheat lgG elevates MBP lgG, α-gliadin 33-mer lgG elevates GAD-65 lgG and MBP lgG, γ-gliadin lgG elevates GAD-65 lgG and MBP lgG, and milk butyrophilin lgG elevates cerebellar lgG, MBP lgG, and MOG lgG (Table 3). We also found that wheat lgA elevates all four brain lgAs, α-gliadin 33-mer lgA elevates MBP lgA, cow’s milk lgA elevates GAD-65 lgA and MBP lgA, α + β-casein lgA elevates MBP lgA, milk butyrophilin lgA elevates GAD-65 lgA, cerebellar lgA, and MBP lgA (Table 4). From Table 5 we found that wheat lgM elevates GAD-65 lgM and MBP lgM, α-gliadin 33-mer lgM elevates GAD-65 lgM, cerebellar lgM and MBP lgM, γ-gliadin lgM elevates GAD-65 lgM, cow’s milk lgM elevates GAD-65 lgM and MBP lgM, α + β-casein lgM elevates cerebellar lgM and MOG lgM, while milk butyrophilin lgM elevates GAD-65 lgM and MBP lgM.

**Table 1:** Results of the simple linear regression between lgG isotypes of food proteins and the brain proteins. The first number in each cell presents corresponding Pearson’s correlation coefficient and the second number presents its *p*-value. Small *p*-value (less than 0.05) indicates statistical significance. Note that *R*^2^ values of these regressions are the squares of the Pearson’s correlation coefficients.

Wheat & Milk Proteins	GAD-65 lgG	Cerebellar lgG	MBP lgG	MOG lgG
wheat lgG	0.0172 0.7314	−0.0078 0.8772	0.1025 0.0404 Significant	−0.0029 0.9534
α-gliadin 33-mer lgG	0.1116 0.0256 Significant	−0.0576 0.2500	0.1456 0.0035 Significant	−0.0024 0.9623
γ-gliadin lgG	0.1251 0.0123 Significant	0.0323 0.5193	0.1479 0.0030 Significant	0.0051 0.9192
cow’s milk lgG	0.0932 0.0626	0.0911 0.0689	0.0595 0.2350	0.0525 0.2949
α + β-casein lgG	0.0763 0.1280	0.0432 0.3897	0.0847 0.0912	−0.0760 0.1296
milk butyrophilin lgG	0.0506 0.3128	0.2680 <0.0001 Significant	0.1642 0.0010 Significant	0.1618 0.0012 Significant

**Table 2:** Results of the simple linear regression between lgA isotypes of food proteins and the brain proteins. The first number in each cell presents corresponding Pearson’s correlation coefficient and the second number presents its *p*-value. Small *p*-value (less than 0.05) indicates statistical significance. Note that *R*^2^ values of these regressions are the squares of the Pearson’s correlation coefficients.

Wheat & Milk Proteins	GAD-65 lgA	Cerebellar lgA	MBP lgA	MOG lgA
wheat lgA	0.2032 <0.0001 Significant	0.1978 <0.0001 Significant	0.2476 <0.0001 Significant	0.2318 <0.0001 Significant
α-gliadin 33-mer lgA	0.0874 0.0984	0.0446 0.3742	0.1757 0.0004 Significant	0.0762 0.1279
γ-gliadin lgA	0.0517 0.3028	−0.0044 0.9300	0.0381 0.4474	0.0166 0.7411
cow’s milk lgA	0.1401 0.0050 Significant	0.0776 0.1213	0.1671 0.0008 Significant	0.0912 0.0683
α + β-casein lgA	0.0346 0.4903	0.0226 0.6521	0.1157 0.0206 Significant	0.0297 0.5537
milk butyrophilin lgA	0.1248 0.0125 Significant	0.0994 0.0469 Significant	0.1483 0.0029 Significant	0.0710 0.1565

**Table 3:** Results of the simple linear regression between lgM isotypes of food proteins and the brain proteins. The first number in each cell presents corresponding Pearson’s correlation coefficient and the second number presents its *p*-value. Small *p*-value (less than 0.05) indicates statistical significance. Note that *R*^2^ values of these regressions are the squares of the Pearson’s correlation coefficients.

Wheat & Milk Proteins	GAD-65 lgM	Cerebellar lgM	MBP lgM	MOG lgM
wheat lgM	0.2032 <0.0001 Significant	−0.0196 0.6962	0.1931 0.0001 Significant	0.0598 0.2328
α-gliadin 33-mer lgM	0.2181 <0.0001 Significant	0.1244 0.0127 Significant	0.2046 <0.0001 Significant	0.0022 0.9653
γ-gliadin lgM	0.1133 0.0234 Significant	−0.0460 0.3587	−0.0062 0.9017	−0.0025 0.9610
cow’s milk lgM	0.2022 <0.0001 Significant	0.0457 0.3620	0.2003 0.0001 Significant	0.0619 0.2170
α + β-casein lgM	−0.0174 0.7289	0.3445 <0.0001 Significant	−0.0211 0.6738	0.3178 <0.0001 Significant
milk butyrophilin lgM	0.2759 <0.0001Significant	−0.0732 0.1442	0.2606 <0.0001 Significant	−0.0131 0.7946

**Table 4:** Results of the simple linear regression between each pair of lgG isotype of the antigens. The first number in each cell presents corresponding Pearson’s correlation coefficient and the second number presents its *p*-value. Small *p*-value (less than 0.05) indicates statistical significance. Note that *R*^2^ values of these regressions are the squares of the Pearson’s correlation coefficients.

Wheat & Milk Proteins	Wheat lgG	α-Gliadin 33-Mer lgG	γ-Gliadin lgG	Cow’s Milk lgG	α + β-Casein lgG	Milk Butyrophilin lgG
wheat lgG	1.0000 0.0000	0.0791 0.1141	0.1486 0.0029 Significant	0.3408 <0.0001 Significant	0.3203 <0.0001 Significant	−0.0683 0.1731
α-gliadin 33-mer lgG	0.0791 0.1141	1.0000 0.0000	0.4871 <0.0001 Significant	0.2058 <0.0001 Significant	0.1535 0.0021 Significant	0.1002 0.0452 Significant
γ-gliadin lgG	0.1486 0.0029 Significant	0.4871 <0.0001 Significant	1.0000 0.0000	0.2009 0.0001 Significant	0.2165 <0.0001 Significant	0.0954 0.0567
cow’s milk lgG	0.3408 <0.0001 Significant	0.2058 <0.0001 Significant	0.2009 0.0001 Significant	1.0000 0.0000	0.4467 <0.0001 Significant	0.1929 0.0001 Significant
α + β-casein lgG	0.3203 <0.0001 Significant	0.1535 0.0021 Significant	0.2165 <0.0001 Significant	0.4467 <0.0001 Significant	1.0000 0.0000	0.0384 0.4449
milk butyrophilin lgG	−0.0683 0.1731	0.1002 0.0452 Significant	0.0954 0.0567	0.1929 0.0001 Significant	0.0384 0.4449	1.0000 0.0000

Next, we performed simple regression analyses between each pair of antigens to check if there are tendencies that the same individuals have high titres to multiple antigens. The summary of the results is presented in Table 4, Table 5, and Table 6. From the tables we see that several pairs of the antigens have significant correlations.

**Table 5:** Results of the simple linear regression between each pair of lgA isotype of the antigens. The first number in each cell presents corresponding Pearson’s correlation coefficient and the second number presents its *p*-value. Small *p*-value (less than 0.05) indicates statistical significance. Note that *R*^2^ values of these regressions are the squares of the Pearson’s correlation coefficients.

Wheat & Milk Proteins	Wheat lgA	α-Gliadin 33-Mer lgA	γ-Gliadin lgA	Cow’s Milk lgA	α + β-Casein lgA	Milk Butyrophilin lgA
wheat lgA	1.0000 0.0000	0.1451 0.0036 Significant	0.1558 0.0018 Significant	0.2994 <0.0001 Significant	0.2667 <0.0001 Significant	0.0701 0.1615
α-gliadin 33-mer lgA	0.1451 0.0036 Significant	1.0000 0.0000	0.1602 0.0013 Significant	0.0468 0.3507	0.0989 0.0482 Significant	0.1067 0.0328 Significant
γ-gliadin lgA	0.1558 0.0018 Significant	0.1602 0.0013 Significant	1.0000 0.0000	0.1037 0.0382 Significant	0.2293 <0.0001 Significant	0.1182 0.0180 Significant
cow’s milk lgA	0.2994 <0.0001 Significant	0.0468 0.3507	0.1037 0.0382 Significant	1.0000 0.0000	0.4489 <0.0001 Significant	0.1502 0.0026 Significant
α + β-casein lgA	0.2667 <0.0001 Significant	0.0989 0.0482 Significant	0.2293 <0.0001 Significant	0.4489 <0.0001 Significant	1.0000 0.0000	0.1396 0.0052 Significant
milk butyrophilin lgA	0.0701 0.1615	0.1067 0.0328 Significant	0.1182 0.0180 Significant	0.1502 0.0026 Significant	0.1396 0.0052 Significant	1.0000 0.0000

**Table 6:** Results of the simple linear regression between each pair of lgM isotype of the antigens. The first number in each cell presents corresponding Pearson’s correlation coefficient and the second number presents its *p*-value. Small *p*-value (less than 0.05) indicates statistical significance. Note that *R*^2^ values of these regressions are the squares of the Pearson’s correlation coefficients.

Wheat & Milk Proteins	Wheat lgM	α-Gliadin 33-Mer lgM	γ-Gliadin lgM	Cow’s Milk lgM	α + β-Casein lgM	Milk Butyrophilin lgM
wheat lgM	1.0000 0.0000	0.3712 <0.0001 Significant	0.0712 0.1553	0.1625 0.0011 Significant	0.0256 0.6094	0.3058 <0.0001 Significant
α-gliadin 33-mer lgM	0.3712 <0.0001 Significant	1.0000 0.0000	0.0099 0.8434	0.2339 <0.0001 Significant	0.0124 0.8055	0.3027 <0.0001 Significant
γ-gliadin lgM	0.0712 0.1553	0.0099 0.8434	1.0000 0.0000	−0.0798 0.1109	0.0118 0.8146	0.0909 0.0694
cow’s milk lgM	0.1625 0.0011 Significant	0.2339 <0.0001 Significant	−0.0798 0.1109	1.0000 0.0000	−0.0147 0.7692	0.2035 <0.0001 Significant
α + β-casein lgM	0.0256 0.6094	0.0124 0.8055	0.0118 0.8146	−0.0147 0.7692	1.0000 0.0000	−0.0376 0.4528
milk butyrophilin lgM	0.3058 <0.0001 Significant	0.3027 <0.0001 Significant	0.0909 0.0694	0.2035 <0.0001 Significant	−0.0376 0.4528	1.0000 0.0000

**Figure 8:**
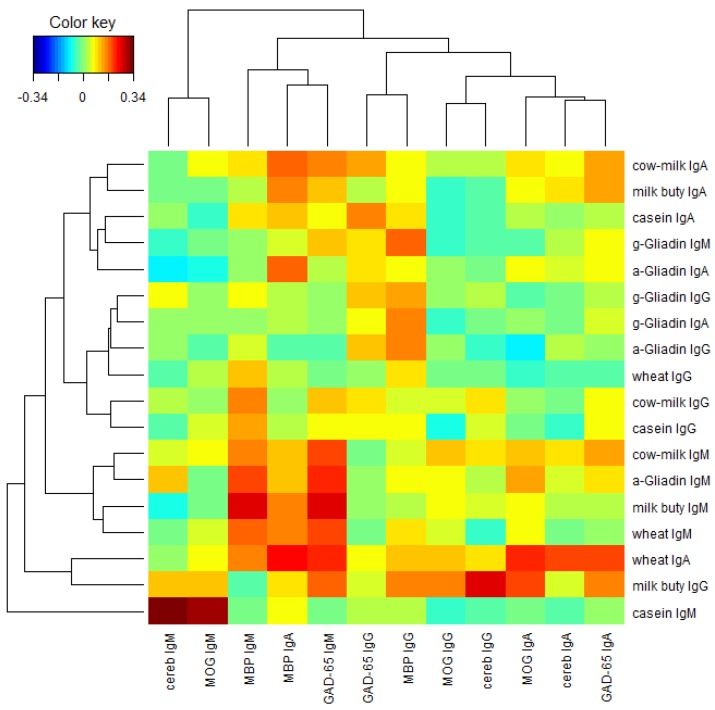
Two-way cluster analysis of the Pearson’s correlation coefficients between the food proteins and the brain proteins.

Finally, we performed a two-way cluster analysis of the Pearson’s correlation coefficients between the food proteins and the brain proteins. Figure 8 presents the result. From Figure 8 we see that lgG, lgA, and lgM isotypes of the brain proteins (columns) are clustered almost separately with the exception of MBP lgA, lgM, lgG, and lgA isotypes of the brain proteins are clustered near the left, middle and right portions respectively in Figure 8. Similar phenomena are observed among the food proteins (rows) with a few exceptions. lgA, lgG, and lgM isotypes of the food proteins are clustered near the top, middle, and bottom portions, respectively, in Figure 8. It shows that the Pearson’s correlation coefficients between the food proteins and the brain proteins are quite different among different isotypes.

## 4. Discussion

The goal of this study was to measure elevations in IgG, IgM, and IgA antibodies against wheat and milk and their major allergens such as α-gliadin, γ-gliadin, α + β-casein, and milk butyrophilin. As wheat and milk proteins and peptides share homology with each other and their antibodies cross-react with neural antigens [13,16,18,20], we wanted to investigate whether or not antibodies detected against gliadin, casein, and butyrophilin contribute to elevation in antibodies against GAD-65, cerebellar, MBP, and MOG.

In our study the serum concentrations and percentage of elevation in antibodies against wheat and cow’s milk in tested populations were very similar. The two-way cluster analysis of the Pearson’s correlation coefficient between the food proteins and brain proteins, as depicted in Figure 8, shows cow’s milk, milk butyrophilin, α + β-casein, α-gliadin, and wheat clustering significantly when cross-referenced with MBP and GAD-65. This simultaneous elevation in wheat, α-gliadin, milk, and α + β-casein may be related to antigenic similarity or cross-reactivity between wheat and milk proteins. It has been shown that there is a high degree of homology or cross-reactivity between bovine α + β-casein and the α-gliadin 33-mer. This homology between milk proteins is demonstrated not only by IgA anti-gliadin antibody immune reactivity with milk proteins [26,31] but also by IgA reactivity to α + β-casein in celiac disease [32] and the induction of a similar local inflammatory reaction after rectal challenge with wheat and milk proteins [33]. This cross-reactivity between gliadin and casein was confirmed in our recent study after application of monoclonal and polyclonal antibodies made against α-gliadin 33-mer and exhibiting strong immune reactivity against milk proteins both by ELISA and dot blot assays [16]. We also demonstrated that mouse or rabbit anti-α-gliadin 33-mer reacted significantly with GAD-65, MBP and cerebellar peptides [16]. Therefore, it was important to look for co-occurrence of IgG, IgM, and IgA antibodies against wheat, milk protein, and neural antigens in human serum. Analysis of data showed that a significant number of specimens with elevation in antibodies against α-gliadin or γ-gliadin peptides exhibited simultaneous elevation in isotype-specific antibodies against GAD-65 and cerebellar peptides (Figure 8). This indicates that cross reactivity of gliadin with GAD-65 and cerebellar contributes only partially to elevation in antibodies against GAD 65 and cerebellar.

In addition, earlier studies have shown that injection of bovine milk protein induced MS-like syndrome in animal model and concluded that consumption of milk products may modulate the pathogenic autoimmune response to MOG peptide sequence 76–87 (IGEGKVTLRIRN) [23]. Furthermore, it was demonstrated that antibody cross-reactivity between the *N*-terminal domain of bovine milk protein with MOG was responsible for the simultaneous detection of butyrophilin-specific antibody, as well as MOG peptide antibody in 34% of MS patients [34]. It was suggested that exposure to common dietary antigens may influence the composition and function of the MOG-specific autoimmune repertoire during the course of MS. Our findings presented in Table 2 showed that only half of the individuals reacting to milk proteins exhibit simultaneous elevations in milk protein as well as MOG- and MBP-specific antibodies (Figure 8).

In the earlier study [34] it was indicated that although the MOG-specific antibody repertoire cross-reacts with multiple butyrophilin peptide epitopes and this pattern of epitope recognition varies from individual to individual, the highest frequency of antibody responses was against peptides spanning amino acid sequences 76–100 of both butyrophilin and MOG, with a match of over 50% (shown below) [34].

MOG_76–100_ IGEGKVTLRIRNVRFSDEGGFTCFF
BTN_76–100_ IAEGSVAVRIQEVKASDDGEYRCFF

In fact, one of our earlier studies investigated the link between immune response to dietary proteins, gliadin, and cerebellar peptides in children with autism. The study showed epitope similarity between gliadin 8-mer (EQVPLVQQ) and cerebellar 8-mer (EDVPLLED) [18]. Anti-gliadin epitopes or anti-cerebellar epitopes reacted almost equally against both gliadin and cerebellar peptides, indicating that this epitope may be responsible for cross-reactive antibody production between gliadin and cerebellar peptides [18]. This difference in epitope recognition was confirmed in the current study by conducting inhibition assays, which demonstrated that while cerebellar peptide was capable of inhibiting cerebellar antibody in all four tested sera by 60%–70%, wheat antigens and α-gliadin inhibited these antibodies by more than 30% in only two out of four of the tested specimens (Figure 6). Similar to these results all sera with high levels of MOG antibodies were inhibited by absorption with MOG, but in only two out of four sera could MOG-specific antibodies be inhibited by milk, milk butyrophilin, and α + β-casein, but not by HSA (Figure 7). With these experiments we demonstrated that cerebellar and MOG peptides, which are known to be important autoantigens in gluten ataxia and MS, can cross-react with wheat and milk proteins. The demonstration of molecular mimicry between α-gliadin, cerebellar peptide, milk butyrophilin, and MOG, and the simultaneous detection of antibodies against these proteins in a small percentage of the general population may have broader implications in the induction of neuroimmune disorders. In these individuals, due to a regulatory defect in mucosal immunity, the consumption of wheat and milk products provides a source of α-gliadin, γ-gliadin, and milk butyrophilin-derived peptides that can cross the gut mucosa to stimulate antigen-specific immune responses both locally in the gut as well as in the periphery. In the majority of the population the cerebellar and MOG normally remains sequestered behind the blood brain barrier (BBB). However, CNS inflammation and BBB breakdown can render the neural tissue antigens accessible to the cross-reactive antibodies and auto-reactive lymphocytes, subsequently resulting in neuroimmune disorders [34,35,36].

## 5. Conclusions

It has been demonstrated that wheat and milk proteins act as molecular mimics of cerebellar peptide and MOG.

This cross-reactivity between α-gliadin and cerebellar peptides, and between milk butyrophilin and MOG peptides, could be responsible for the simultaneous detection of antibodies against these molecules in a small percentage of tested blood samples.

The pathophysiological consequences of molecular mimicry involving wheat and milk with human tissue antigens are difficult to predict, as is the detection of antibodies against them in human sera. This is because they can be influenced by multiple factors, including an individual’s genotype, the timing and level of exposure, and the health of the gut and blood brain barriers, and as such these complex interactions deserve further study.
